# When Does the Cytokine Storm Begin in COVID-19 Patients? A Quick Score to Recognize It

**DOI:** 10.3390/jcm10020297

**Published:** 2021-01-15

**Authors:** Stefano Cappanera, Michele Palumbo, Sherman H. Kwan, Giulia Priante, Lucia Assunta Martella, Lavinia Maria Saraca, Francesco Sicari, Carlo Vernelli, Cinzia Di Giuli, Paolo Andreani, Alessandro Mariottini, Marsilio Francucci, Emanuela Sensi, Monya Costantini, Paolo Bruzzone, Vito D’Andrea, Sara Gioia, Roberto Cirocchi, Beatrice Tiri

**Affiliations:** 1 Clinical Infectious Disease, Department of medicine, St. Maria Hospital, 05100 Terni, Italy; m.palumbo@aospterni.it (M.P.); giulia.priante1989@gmail.com (G.P.); l.martella@aospterni.it (L.A.M.); lavitrinity@libero.it (L.M.S.); francesco.sicari@studenti.unipg.it (F.S.); c.vernelli@aospterni.it (C.V.); c.digiuli@aospterni.it (C.D.G.); tiri.beatrice@gmail.com (B.T.); 2 Department of General Surgery, Royal Perth Hospital, Perth 6000, Australia; sherm.k@live.com; 3 Hematology and Microbiology Laboratory, St. Maria Hospital, 05100 Terni, Italy; p.andreani@aospterni.it (P.A.); a.mariottini@aospterni.it (A.M.); 4 Department of General and Oncologic Surgery, St. Maria Hospital, 05100 Terni, Italy; m.francucci@aospterni.it; 5 Department of Critical Care Medicine and Anesthesiology, St. Maria Hospital, 05100 Terni, Italy; e.sensi@aospterni.it; 6 Pharmacy Unit, St. Maria Hospital, 05100 Terni, Italy; m.costantini@aospterni.it; 7 Department of General and Specialist Surgery “Paride Stefanini”, 00185 Rome, Italy; paolo.bruzzone@uniroma1.it; 8 Department of Surgical Sciences, Sapienza University of Rome, 00161 Rome, Italy; vito.dandrea@uniroma1.it; 9 Legal Medicine, University of Perugia, 06123 Perugia, Italy; sara.gioia@unipg.it; 10 Department of General and Oncologic Surgery, University of Perugia, St. Maria Hospital, 05100 Terni, Italy; roberto.cirocchi@unipg.it

**Keywords:** COVID-19, cytokine storm, ARDS

## Abstract

Severe acute respiratory syndrome coronavirus 2 (SARS-CoV-2) is the virus that is responsible for coronavirus disease 2019 (COVID-19), which has rapidly spread across the world, becoming a pandemic. The “cytokine storm” (CS) in COVID-19 leads to the worst stage of illness, and its timely control through immunomodulators, corticosteroids, and cytokine antagonists may be the key to reducing mortality. After reviewing published studies, we proposed a Cytokine Storm Score (CSs) to identify patients who were in this hyperinflammation state, and at risk of progression and poorer outcomes. We retrospectively analyzed 31 patients admitted to Infectious Disease Department in “St. Maria” Hospital in Terni with confirmed SARS-CoV-2 infections, and analyzed the “CS score” (CSs) and the severity of COVID-19. Then we conducted a prospective study of COVID-19 patients admitted after the definition of the CSscore. This is the first study that proposes and applies a new score to quickly identify COVID-19 patients who are in a hyperinflammation stage, to rapidly treat them in order to reduce the risk of intubation. CSs can accurately identify COVID-19 patients in the early stages of a CS, to conduct timely, safe, and effect administration of immunomodulators, corticosteroids, and cytokine antagonists, to prevent progression and reduce mortality.

## 1. Introduction

Severe acute respiratory syndrome coronavirus 2 (SARS-CoV-2) emerged for the first time in Wuhan, China, in December 2019. The virus is responsible for coronavirus disease 2019 (COVID-19), which has rapidly spread across the world. The World Health Organization (WHO) declared COVID-19 a pandemic in March 2020 [1].

As of August 21, 2020, the WHO reported 22.536.278 confirmed cases of coronavirus disease 2019 (COVID-19) worldwide. Ref. [2] As of August 21, there have been 256,118 confirmed cases with 35,418 deaths for COVID-19 in Italy [3].

Patients with coronavirus disease 2019 (COVID-19) exhibited a broad spectrum of clinical symptoms. The disease often begins with flu-like symptoms, such as fever, fatigue, dry cough, shortness of breath, headaches, chest tightness, chest pain, and muscle pain. Some patients also present with nausea, vomiting, and diarrhea [4].

Complications during COVID-19 can include severe infections, such as pneumonia and acute respiratory distress syndrome (ARDS); it can also lead to multiple organ dysfunction syndrome (MODS) or even death [5]. Initially recognized as an ARDS disease, it was soon realized that heart, brain, and kidney involvement was also common in COVID-19 [6].

A recent clinical stage proposal categorized COVID-19 as Stage I (mild), Stage II (moderate) with pulmonary involvement without hypoxia (IIa) and with hypoxia (IIb); and Stage III—characterized by ARDS, with a progression, due to a gradual decrease in the virological phase and an increase in the inflammatory one [7].

The cytokine storm (CS) syndrome represents the most feared and serious complication of COVID-19 patients due to an excessive immune response reaction to the virus triggered by inflammatory cell infiltration in the lungs, activation of T-helper 1 reactions, and abundant release of proinflammatory cytokines into the circulation [8]. The CS syndrome led to MODS and disseminated intravascular coagulation (DIC) and most studies have demonstrated the presence of venous thromboembolism and microthrombi in arterioles and venules in COVID-19 patient autopsies [9]. Therefore, some authors suggest that timely control of this CS in its early stage, through immunomodulators, corticosteroids, and cytokine antagonists, is key to reducing the mortality rate of these patients [7].

However, there has yet to be any suggestions or observations on how to recognize when this CS begins.

The aim of our study is to report a new smart score that can identify at-risk patients so they may be quickly treated and the progression of COVID-19 may be prevented.

## 2. Materials and Methods

We planned a retrospective analysis of data on COVID-19 patients admitted to the Infectious Disease Department of Terni Hospital, Italy, from March 11 to April 15, 2020. The inclusion criteria applied to participants were: any gender, > or = 18 years old, hospitalized due to clinical/instrumental diagnosis of pneumonia, virological diagnosis of SARS-CoV-2 infection (CFX96 Touch Real-Time PCR Detection System; ^®^Bio-Rad).

Patients were assessed daily and staged according to the three stage criteria by Siddiqi H.K. et al. [8], which was then recorded in patient charts.

The staging criteria (by Siddiqi H.K. et al.) organized COVID-19 infections into the following three stages:

- Stage I (mild): early infection where the viral response phase is predominant, characterized by non-specific symptoms, such as malaise, fever, or cough.
- Stage II (moderate): pulmonary involvement without hypoxia (IIa) or with hypoxia (IIb), as defined by shortness of breath or a PaO2/FiO2 ratio ≤ 300. In this stage there were abnormalities on chest imaging. In Stage IIa, the viral response phase is predominant while the inflammation phase is beginning. In Stage IIb, the viral response phase decreases and the host inflammatory response increases.
- Stage III (systemic hyperinflammation): the most severe stage of illness manifests as an extra-pulmonary systemic hyperinflammation syndrome characterized by ARDS, shock, and cardiac failure.

**Figure 1:**
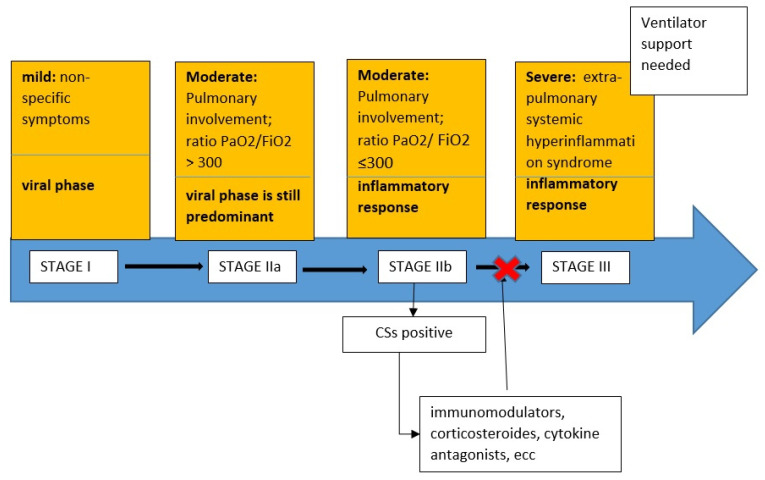
Cytokine Storm Score (CSs) score identifies coronavirus disease 2019 (COVID-19) patients who are doing a cytokine storm, and identifies the timing of correct administration of immunomodulators, corticosteroids, and cytokine receptor antagonists to prevent the progression of stage III COVID-19.

After careful revision of published studies, we proposed a Cytokine Storm Score (CSs) to identify patients who were in this hyperinflammatory state and at risk of progression and poorer outcomes, such as invasive ventilatory support. (Figure 1)

Lymphopenia was chosen as the first main criterion for this CSs because the dysregulated immune response in COVID-19 produces an immune suppressive effect observed through the reduction of peripheral CD4 T and CD8 T cells [10]. Some studies suggest that SARS-CoV-2 may be able to infect T cells directly [11,12]; however, the exact mechanism causing lymphopenia remains unknown. Nonetheless, it is associated with severity of COVID-19. Refs. [13,14,15] Lymphopenia occurs when your lymphocyte count is less than 1000 × 10^3^/mmc, as reported in five other studies that observed lymphocyte counts in patients with COVID-19 [14,15,16] (Table 1).

In patients with lymphopenia, further laboratory evaluations were performed. In COVID-19 patients, the elevation of other laboratory biomarkers, such as D-dimer, lactate dehydrogenase (LDH), ferritin, and C-reactive protein (CRP) were shown to indicate patients with poor prognosis at an early stage of the disease [14,17,18]. Recent research showed that severe COVID-19 patients had lymphopenia and proinflammatory cytokine storms (e.g., high level of CRP, LDH) [19,20].

D-dimer is a fibrin degradation product, a small protein fragment present in the blood after a blood clot is degraded by fibrinolysis. High D-dimer levels appear to be correlated with severity of disease; the level of D-dimer reported is variable, but the median value of the D-dimer level reported in literature, correlated with severity of COVID-19, was about 1000 ng/mL [21,22,23].

D-dimer greater than 1000 ng/mL identified COVID-19 patients with poor prognosis at an early stage and a prospective study reported that the median D-dimer level in COVID-19 patients admitted to Intensive Care Unit (ICU) was 1000 ng/mL [24].

The lactate dehydrogenase (LDH), a marker of tissue damage, was also associated with severe COVID-19 patients [14,25,26]. The mean value in patients with mild disease was 305.6 U/L versus 542.5 U/L in extremely severe ones. In another study, the mean value in 90.9% of severe patients was >300 U/L [15].

Ferritin is an acute-phase-protein exhibiting elevated serum concentrations in various inflammatory diseases. Cytokine storm syndrome and elevated ferritin levels, considered as hyperinflammation, were reported in severe COVID-19 patients [27]. Multiple publications show that higher ferritin levels, along with other pro-inflammatory markers, are correlated with worse outcomes [15,16,28,29,30,31]. Ferritin could exert a pathogenic role in these diseases rather than just being the result of hyperinflammation. In fact, ferritin synthesis is mediated not only by iron availability, but also by interleukin (IL)-1, IL-6, and Tumor Necrosis Factor (TNF), with overexpression of pro-inflammatory cytokines, thus becoming part of a vicious loop [32].

Therefore, the elevation of serum ferritin was suggested as a predictor of COVID-19 mortality, but is less understood [27]. The mean value reported in severe patients was different, some reported 440 ng/mL, others >800, and others reported 614 ng/mL [15,33,34].

In a recent meta-analysis, the biggest difference for severity was for ferritin at 423.13 ng/mL (281.4–582.85) [25].

Serum CRP levels were considered independent risk factors for poor prognosis in COVID-19 [13].

The CRP cut-off of 10 mg/dL in pneumonia was associated with mortality at 30 days, mechanical ventilation, and pneumonia complications [35,36].

Lymphopenia was chosen as the first main criterion for this CSs. If at least two of the serum levels of D-dimer, ferritin, or lactate dehydrogenase (LDH) were deranged, as defined in Table 1, the CSs was positive, and a hyperinflammatory stage of COVID-19 was suspected.

In patients with lymphopenia and derangement of only one of D-Dimer, ferritin, or LDH, then the C-reactive protein (CRP) was measured. A CRP greater than 10 mg/dL was considered positive, and, together with lymphopenia, and derangement of D-Dimer, ferritin, or LDH, would result in a positive CSs (Table 1).

**Figure 2:**
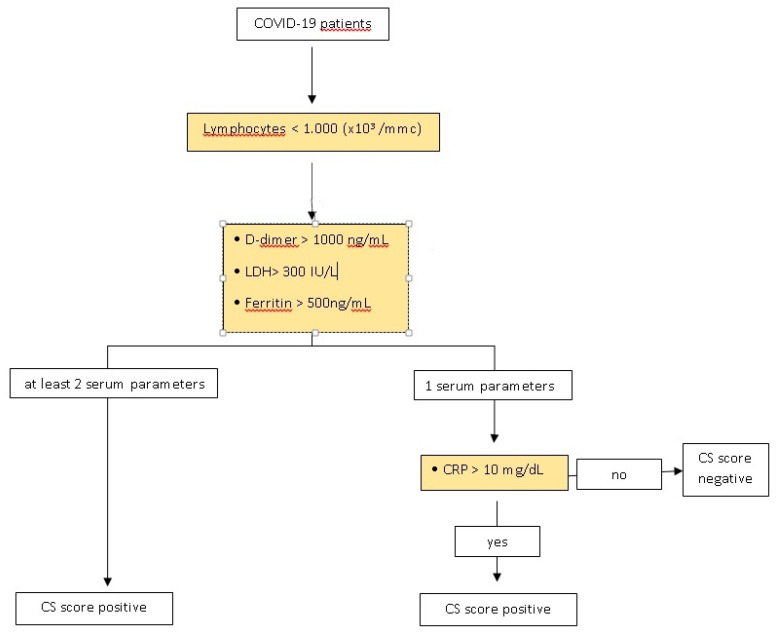
CS score’s algorithm.

The algorithm of CS score was reported in Figure 2.

COVID-19 patients performed routine biochemistry and we were able to calculate the CS score.

Since April 15, we conducted a prospective study, enrolling all patients admitted to our clinic with the same inclusion criteria: any gender, > or = 18 years old, hospitalized due to clinical/instrumental diagnosis of pneumonia, virological diagnosis of SARS-CoV-2 infection (real-time PCR).

Each day, we analyzed the CSscore for these patients. Based on therapeutic management evidence, which changed during the months of the pandemic, in patients with a positive CSscore, we started administration of immunomodulators (tocilizumab), corticosteroids, or both. In particular, after 17 June 2020, when the Italian Drug Agency (AIFA), suspended the use of tocilizumab in the absence of confirmed clinical evidence of the use of immunomodulators, patients were only treated with corticosteroids.

## 3. Results

Between 1 March and 15 April 2020, 31 patients were admitted to Infectious Disease Department with confirmed SARS-CoV-2 infection. There were 19 males (61%) and 12 females (39%); the average age of the whole group was 61.35 years (with an age range of 37 to 92 years).

Ten patients (32%) had evidence of radiological pulmonary involvement (either with plain film or computed tomography) without hypoxia (SaO2 > 94%) and were classified as Stage IIa. The average age in this group was 56.4 years (range) and the gender distribution was equal.

Twenty-one patients (68%) had pulmonary involvement with hypoxia (SaO2 ≤ 93%) and were classified as Stage IIb. The average age in this group was 63.7 years (range) and the ratio of male to female was 2:1. Ten patients (48%) in Stage IIb did not progress to Stage III while 11 (52%) progressed to requiring invasive ventilator support.

A retrospective analysis of these 31 patients demonstrated all ten patients in Stage IIa had a negative CSs. The patients in Stage IIb, who did not progress, also had a negative CSs negative in 100% of cases (*n* = 10). However, patients with Stage IIb infection who progressed had a positive CSs in 100% of cases (*n* = 11) (Table 2).

The time between intubation and recognition of a positive CSs was also analyzed, with a mean time of 37 ± 23.14 h (Table 3).

Based on the results reported in the retrospective analysis, we hypothesized the therapeutic diagnostic process illustrated in Figure 1.

From April 15 to August 31, a total of 17 patients with COVID-19 were hospitalized in our clinic: 15 males (88.2%) and 2 females (11.8%); the average age of the whole group was 56.9 years (range 20–78 years).

Ten patients (58.8%) had evidence of radiological pulmonary involvement (either with plain film or computed tomography) without hypoxia (SaO2 > 94%) and were classified as Stage IIa. The average age in this group was 49.5 years (range 20–71); 9 were male (90%).

**Figure 3:**
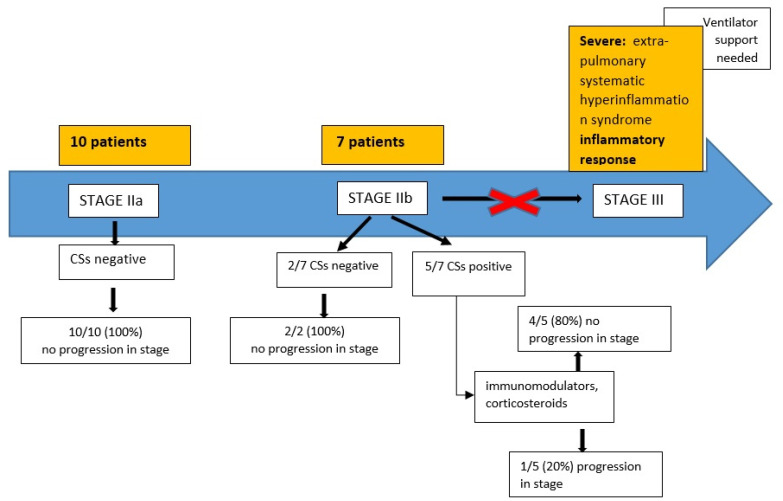
Patients admitted to Infectious Diseases Department with confirmed SARS-CoV-2 infection between April 15 and August 31.

Seven patients (41.2%) had pulmonary involvement with hypoxia (SaO2 ≤ 93%) and were classified as Stage IIb. The average age in this group was 67.5 years (range 54–78); 6 were male (85.7%). Data are reported in Figure 3.

Five out of seven patients (71.4%) in Stage IIb had a positive CSs. Two out of five (40%) were treated with tocilizumab (8 mg/Kg on day 1, with a second administration after 12 h) plus corticosteroids (dexamethasone 20 mg/day for 5 days, then 10 mg/day for 5 days); three out of five (60%) only received corticosteroids.

Three out of five patients after treatment had a stage regression, from stage IIb to stage IIa.

One patient remained stable in stage IIb without progression, while one patient evolved to stage III and required a non-invasive ventilator support.

## 4. Discussion

This is the first study that proposed and applied a new score to quickly identify COVID-19 patients who were in a hyperinflammation stage, and to rapidly treat them to reduce the risk of intubation (ventilator invasive support).

It has long been believed that cytokines play an important role in immunopathology during viral infection. A rapid and well-coordinated innate immune response is the first line of defense against viral infections. However, dysregulated and excessive immune responses cause damage to the human body [37,38]. In severe COVID-19 infected individuals, interleukin (IL)-6, IL-10, and TNF-α are significantly higher, and it is known that proinflammatory cytokines and chemokines contribute to the occurrence of ARDS [39,40]. Most authors suggest this “cytokine storm” in COVID-19 is responsible for progression to a more severe stage of illness [41,42], and its early control is key to reducing mortality [1]. The treatments proposed in various studies have included corticosteroid therapies (commonly used to suppress inflammation), Janus kinase (JAK) inhibitors (IL-1 family antagonists) or IL-6 receptor blockade (IL-6 antagonists), and TNF blockers. The randomized controlled COVACTA trial NCT04320615 (A Study to Evaluate the Safety and Efficacy of Tocilizumab in Patients With Severe COVID-19 Pneumonia)failed to meet its primary end point of improved clinical status [43], but evidence in the literature, instead, reports efficacy with tocilizumab [44,45,46]. Care should be taken in interpreting data from the COVACTA study, one consideration is that something must be done about the timing of the administration, because the immunosuppression (with glucocorticoids or IL-6 blockade) needs to intervene when the immune response makes the patient sick. The COVACTA trial missed clinically relevant differences between patient groups [47]. In fact, in studies reporting efficacy of tocilizumab, the administration was related to the presence of hyperinflammatory syndrome [45,46]. The RECOVERY trial NCT04381936 (Randomised Evaluation of COVID-19 Therapy) results show a clear benefit of dexamethasone in patients who require respiratory support, but not in patients who do not require respiratory support. This suggests that the timing of anti-inflammatory treatments, in relation to the stage of the disease, is important [48].

In our study, the CSscore seems to identify the timing of administration of tocilizumab (TCZ) and or dexamethasone. Indeed, 80% of patients with positive CSs, who received TCZ and/or dexamethasone, did not have progression in stage. Timing of therapy is important for COVID-19 patients potentially entering this cytokine storm. Premature administration of corticosteroid, Janus kinase (JAK) inhibitors, Il-6 receptor blockade (IL-6 antagonists), and TNF blockers, inhibits the body’s initial immune defense mechanism, leading to adverse effects. Conversely, delayed administered also leads to worse outcomes, such as intubation.

Metha et al. proposed a score (HScore) to identify a subgroup of patients where immunosuppression could improve mortality [34]. The HScore was validated for secondary hemophagocytic lymphohistiocytosis (sHLH), which is an hyperinflammatory condition characterized by hypercytokinemia and multi-organ failure. The HScore was based on 25 patients with sHLH due to an infectious state (not COVID-19 related) and needed the bone marrow aspirate, which is very difficult to perform in common clinical practices [34,49].

Other studies analyzed the laboratory differences between severe and moderate cases of COVID-19 to identify any risk factors or patterns that led to increased severity. Ruan et al. analyzed the difference in mortality between two subgroups of COVID-19 infections; a discharge group and a death group. They observed significant differences in white blood cell counts, C-reactive protein (CRP), and IL-6, and concluded that COVID-19 mortality may be due to a virus-related “cytokine storm”. Ref. [16] Chen et al. evaluated the difference between severe and moderate COVID-19. Lymphocyte counts were significantly lower in severe cases (lymphocyte count < 0.8 × 10^9^) while LDH, CRP, and D-dimer levels were markedly higher in severe cases compared to moderate cases [15]. A meta-analysis by Zheng et al. concluded that LDH >245 U/L and D-dimer > 0.5 mg/L predicted deterioration with COVID-19. Ref. [14] Zhou et al. reported that D-dimer> 1000 mg/mL on admission is related to an increased likelihood of death in hospital [19]. Recently, Ibanez et al. reported in a prospective study that the median D-dimer level in COVID-19 patients admitted to ICU was 1000 ng/mL [24]. The correlation between the high level of CRP and the severity of COVID-19 disease has been well established in the literature, as is the combination of lymphopenia and CRP concentration as a sign of severe infection [50,51,52,53,54].

Recently Wang K. et al. reported that CRP was the most important predictor for the mortality of patients with COVID-19 [55].

However, laboratory results in COVID-19 patients showed great variability between studies, because, in our judgment, there were many differences in the classification of COVID-19 patients, and in the definition of the subgroups analyzed (severe vs. non-severe; survivors vs. non-survivors; etc.).

All Stage IIa patients had a negative CSs score and did not progress, which seems to correlate with Siddiqi H.K. et al. and their idea that, in this stage of disease, the viral response phase is predominant, and the reactive inflammation is low [7]. In Stage IIb patients, it appears a transition from viral response to host inflammatory response occurs, with some patients progressing to Stage III and others not. Our CSs score was positive in all patients who progressed to ARDS and required ventilatory support within an average time of 37 ± 23.14 h. Where CSs was negative, these Stage IIb patients remained stable and did not progress, suggesting our CSs can be used to predict those who may benefit from early intervention to prevent progression.

Moreover, in the group of patients admitted after April 15, we observed that those in stage IIa had all a negative CS score and had no progression in stages, such as in the retrospective analysis.

With experience from the previous months, we used the CS score to evaluate the initiation of therapy in stage IIb patients. CS score was positive in 5/7 stage IIb patients. At the time of the positive score, patients started therapy with immunomodulators, corticosteroids, or both, depending on the evidence on therapeutic management that changed during the months of the pandemic. In particular, on May 17, the Italian Drug Agency (AIFA) suspended the use of tocilizumab after the publication of a study showed that early administration of tocilizumab in patients with COVID-19 pneumonia does not provide any relevant clinical benefit to patients [56]. Four of the 5 patients (90%) after treatment had a stage regression from stage IIb to stage IIa, confirming that the CS score can be useful to identify the stage of hyperinflammation and prevent progression.

From a careful reading of the RECOVERY trial, it is not clear what the indication is for the use of oxygen, i.e., it takes into consideration a large slice of the population that needs O2 therapy [48]. The WHO published guidelines on corticosteroids for COVID-19 (2 September 2020), in which it suggests the use of corticosteroids in critical and severe COVID-19, defining severe COVID-19 as oxygen saturation <90% on room air, respiratory rate >30 breaths per minute in adults, and sign of severe respiratory distress. They warn that patients with a saturation between 90 and 94% breathing room air should not be offered systematically corticosteroids, but clinicians must use their judgment to give them corticosteroids [57].

Moreover, recent Infectious Disease Society of America (IDSA) guidelines on the treatment and management of patients with COVID-19 “suggest against glucocorticoids for patients with COVID-19 without hypoxemia requiring supplemental oxygen” [58].

Therefore, in our opinion, our score would be useful to identify patients in O2 therapy who need corticosteroids therapy, to better define the population that could really benefit from it.

Future studies will be needed to evaluate patients in O2 therapy who received corticosteroids with a positive CSscore, versus a CSnegative, in order to evaluate the outcome.

The biggest limitation to our study is the small sample size; therefore, it will be necessary to apply it to a larger sample of patients, and in a prospective manner, to validate the CS score.

## 5. Conclusions

Our study demonstrates that a new CSs could be used to accurately identify the onset of hyperinflammation in COVID-19 patients, which may ultimately help prevent progression to a more severe stage. This may then lead to timely, safe, and effective administration of therapies to prevent progression and potentially reduce mortality. Further studies, with larger sample sizes, would be useful to validate this score.

## Figures and Tables

**Table 1:** Definition of Cytokine Storm score (CSs); (LDH: Lactate dehydrogenase; CRP: C-reactive protein).

Cytokine Storm Score (CSs)	
**At Lymphocytes < 1000 (×10^3^/mmc):**	
And at least 2 of the following parameters:	**positive**
D-dimer > 1000 ng/mL	
LDH> 300 IU/L	
Ferritin > 500 ng/mL	
**OR**	
**At Lymphocytes < 1000 (×10^3^/mmc):**	
And at least 1 of the following parameters:	**positive**
D-dimer > 1000 ng/mL	
LDH> 300 IU/L	
Ferritin > 500 ng/mL	
AND CRP > 10 mg/dL	

**Table 2:** Retrospective analysis of patients admitted to Infectious Disease wards with confirmed severe acute respiratory syndrome coronavirus 2 (SARS-CoV-2) infection.

	Total Cases	Average Age	Male/Female	CSs	Progression in Stage
COVID-19 patients	31	61.35	19/12	11/31 (35.4%) positive	11/31
Stage IIa	10/31 (32%)	56.4	5/5	10/10 (100%): negative	0/10
Stage IIb	21/31 (68%)	63.7	14/7	10/21 (48%): negative	0/10
**Stage III**				11/21 (52%): positive	11/11 (100%)

**Table 3:** Time (hours) from CSs positive and intubation in patients with Stage IIb progressing to Stage III.

Patients Stage IIb Who Progressed (*n* = 11)	Time (Hours) from CSs Positive and Intubation
2	12 h
4	24 h
4	48 h
1	96 h

## Data Availability

The data presented in this study are available on request from the corresponding author.
